# Supplementary material for: Exploring the prediction of emotional valence and pharmacologic effect across fMRI studies of antidepressants
Source: Neuroimage Clin. 2018 Aug 11;20:407–14. doi: 10.1016/j.nicl.2018.08.016 (PMC6096053; doi:10.1016/j.nicl.2018.08.016)

**Supplementary Materials for:** Exploring the prediction of emotional valence and pharmacologic effect across fMRI studies of antidepressants.

**Authors:**

Daniel Barron^1,2^, Mehraveh Salehi^3,4^, Michael Browning^5,6^, Catherine J Harmer^5,6^, Todd Constable^7-9^, Eugene Duff^10,11^

1. Yale University School of Medicine, New Haven, CT, USA
2. Yale University Department of Psychiatry, New Haven, CT, USA
3. Department of Electrical Engineering, Yale University, New Haven, CT, USA
4. Yale Institute for Network Science, Yale University, New Haven, CT, USA
5. Oxford University Department of Psychiatry, Oxford, United Kingdom
6. Oxford Health NHS Trust, Oxford, UK
7. Interdepartmental Neuroscience Program, Yale University School of Medicine, New Haven, CT, USA
8. Department of Radiology and Biomedical Imaging, Yale University School of Medicine, New Haven, CT, USA
9. Department of Neurosurgery, Yale University School of Medicine, New Haven, CT, USA
10. Oxford University, FMRIB, Nuffield Department of Clinical Neurosciences, Oxford, United Kingdom
11. Oxford University, Department of Paediatrics

**SUPPLEMENTARY METHODS**

**Preprocessing**

Standard preprocessing and mapping analysis were employed using tools from FMRIB’s Software Library (FSL) package (http://fsl.fmrib.ox.ac.uk/fsl/fslwiki/). Preprocessing included brain extraction (BET), head motion correction (McFLIRT), and a 6mm FWHM smoothing kernel (SUSAN) (1). Pre-whitening was used on all scans. A high-pass filter cutoff of 100s was used. No scans showed an excess of 3mm of motion across frames.

**Study-level analyses:**

For each study, the pre-processed functional data were fit with a temporal model composed of separate regressors for each of the different face presentations, which varied depending on the study. The amplitude of the regressors was kept consistent across all studies to make them comparable. Each study used a variant of the emotional faces task (masked and unmasked presentation of happy, fearful, and sad faces). Only those face presentations that were consistent across studies were contrasted: fearful faces versus fixation, happy faces versus fixation, happy and fearful faces versus fixation and fearful faces versus happy faces. When possible, unmasked faces were used, see Table 1.

The FSL FMRI Expert Analysis Tool (FEAT) was used for general linear modeling (GLM) (1). Each dataset was spatially smoothed with a 6mm FWHM kernel. Subject-level contrast of parameter estimate (COPE) maps for each contrast (e.g. happy versus fixation) were produced in native patient space. FILM prewhitening was used to create these COPE maps. These COPE maps were used in subsequent classification analyses, as described below. These COPE maps were registered to MNI-152 space using transforms based on high-resolution structural images using the FSL linear and non-linear registration tools FLIRT (full search, 7DOF) and FNIRT (full search, 7 DOF, warp resolution 10mm) (1). Once in MNI-152 space, COPE maps were fed into the higher-level study and multi-study GLM analyses.

Study-level effect maps were generated from session-level response maps using a second-level paired GLM that modeled consistent pair-wise differences between drug and placebo sessions. The study-level models included a regressor modeling the average difference between drug and placebo session responses. Group-level effect maps were generated across all studies, representing a third level of analysis. See Figure 1 for an illustration of the analysis pipeline.

**Quality Assurance (QA)**

The validity of any meta-analysis rests on the validity of the included datasets. Therefore in addition to standard quality control procedures built into the FSL software tools (1), we implemented an additional, meta-analytic assessment designed to identify issues producing inadequate measured changes in BOLD activity between task and rest; these issues non-responsive subjects, incorrect timing specification, inaccurate hemodynamic models, or non-optimal filtering, that are unlikely to be identified from low-level data assessment.

The assessment focused on regions that were significantly activated or deactivated across the other studies in a GLM-based meta-analysis performed using the FSL FEAT software (33). Studies that showed significantly lower responses compared to others were flagged as potentially problematic (significant regions here identified by the spatial thresholding described in the main text under “Statistical analysis”). Flagged studies were subject to further assessments to determine the source of the unexpectedly low values. Model fits were compared to average responses, to identify modeling errors such as incorrect timing specification, inaccurate HRF models, or non-optimal filtering. If other stimuli were used, these were investigated to determine whether responses to these stimuli were affected. Any problems that were identified were corrected, and all analyses were repeated with the corrected data. Note that this assessment was entirely independent of the presence or absence of an anti-depressent effect.

**Machine Learning Method**

Cognitive models of depression suggest that patients process negative relative to positive stimuli differently from non patients, and that these cognitive processes are causative in the illness. Therefore a contrast looking at the emotional processing circuit activation to negative vs. positive faces may be able to identify illness specific signature and how the brain’s emotional circuits change in response to treatment.

A desired feature of our machine learning algorithm was an ability to identify which features were most predictive of a specific outcome, i.e. which features were most predictive of emotional valence or pharmacologic effect. This means we needed an algorithm that, following training of the classifier, could map feature weightings back onto specific features and, therefore, back into anatomical brain space. While there are many options, we chose a gradient boosting machine (GBM) algorithm due to its robustness to outliers and ability to map features back into anatomical brain space (2) .

We performed 2 types of classifications:

1. Emotional Valence. This analysis determined whether and where a signal for emotional valence was consistent enough across subjects and studies to discriminate Fear and Happy face visual conditions. We assessed the performance of our classifier with two different feature inputs to determine the impact of inter-subject variability. The first subtracted fear and happy responses within-subject, to account for average differences in visual responses across subjects. The second set of inputs were the separate fear versus fixation COPE files and happy versus fixation COPES. By not normalizing the subject responses, the latter inputs simulate the drug contrasts, which could only be assessed across subjects due to their parallel groups design.
2. Pharmacologic Effect. This analysis used contrasts between Fear and Happy conditions (FvH COPE files) to discriminate patients with drug or placebo protocols within and across studies. We further observed whether this signal was consistent across antidepressant dose, frequency, and duration.

We tailored the predictive pipeline and cross validation strategy based on the level of the classification performed. There were 4 levels of classification performed:

1. within-study classification: when subjects within one study were considered (i.e. to determine the reliability of effects within individual studies), the classifier was trained on all but two subjects and then tested on those held out subjects in an iterative fashion, until the classifier was tested on all subjects. In each case, the held out subjects were balanced for classification group (one subject was held out for testing from each group). In cases where groups had an unequal number of subjects, the training group was also balanced, meaning equal numbers from each group were used to train the classifier. This was a custom code created in python.
2. across-study classification: when subjects across two studies were considered (i.e. to assess the similarity of trained classifiers across individual studies), the classifier was trained on one study and tested on the other.
3. across-all-subjects classification: when subjects across all studies were considered, to determine our ability to build classifiers that generalize across subjects, the classifier was trained on all but two subjects and then tested on those held out subjects in an iterative fashion, until the classifier was tested on all subjects. The training and testing groups were balanced in the same manner explained in (i).
4. across-all-studies classification: when subjects across all studies were considered (i.e. to assess how a classifier trained on all studies performed on a held out study), the classifier was trained on all studies except one and tested on that held out study.

In all pipelines, a GBM classifier was applied to p-dimensional feature space, where p is the number of nodes in the parcellation (described in further detail in Feature Reduction, below).

Classification accuracy was calculated as the number of correct predictions divided by the number of all predicted outcomes (i.e. the size of the test set, *n*). Accuracy ranges were calculated using the Wilson-Score confidence interval (alpha=0.05, sample size as indicated). P-values for accuracies were calculated based on a binomial distribution, indicating a null hypothesis that the specific accuracy score could have resulted randomly based on the number of samples (i.e. *n;* chance=50%). Feature weightings were averaged across all iterations and normalized to 1 by dividing by the highest reported weighting score. These scores were computed per feature and mapped voxel-wise back into MNI-152 space so they could be shown in anatomical space. These steps took place in MATLAB.

**Feature Reduction**

A voxel-wise analysis (wherein each voxel represented a separate feature) will produce a number of features that far outnumbers the number of subjects, leading to the potential for overfitting. To reduce the number of features, we used the Shen 268-node resting-state fMRI atlas, defined by a group-wise spectral clustering algorithm applied to a 45 subject dataset.(3; 4) The Shen atlas was transformed from MNI-152 space into native patient space using transforms described above and was used to define features in native patient space using subject-level COPE files for each contrast. Parameter values within each parcel were averaged to produce 268 features for the classifier.

SUPPLEMENTARY RESULTS

**Emotional Valence: Happy from Fearful Face Classification**

The successful classification results when trained on healthy subjects and tested on subjects with dysphoric trait or MDD (and vice-versa) indicate some level of similarity across these groups, meaning that informative and transferrable features are captured from one group to reliably predict another. However, since these are clinically distinct populations, we considered the classifier weightings (i.e. the importance assigned to each feature) in two groups: weightings trained on healthy subjects and weightings trained on dysphoric and MDD subjects. The weighting scores were not strongly correlated across clinical groups (rho = 0.096, p=0.12), indicating a difference in the underlying signal across these groups. For healthy subjects, the strongest 10% parcel weightings were found in regions including the left medial and middle frontal gyrus, right thalamic pulvinar, left cingulate, left middle temporal, and bilateral lingual gyri. Overall, these weightings were lateralized to the left hemisphere (see Supplementary Materials). For the dysphoric and MDD subjects, the strongest 10% parcel weightings were found in regions including the bilateral amygdalae, left middle and inferior frontal gyrus, left cingulate, right insula, left fusiform, left inferior parietal. Overall, the strongest weightings for the dysphoric and MDD studies were lateralized to the right hemisphere, however the majority were in the left hemisphere (see Supplementary Materials). For both of these groups, the strongest 10% of parcel weightings overlapped in the right fusiform gyrus. See Figures 3A and 3B for illustrations in MNI-152 space.

**Pharmacologic Effect: Drug from Placebo Classification**

Because the results across all classifications was poor, the location of highly weighted parcels scores were not considered sensitive or specific predictors of pharmacologic effect and so are not presented here.

**References:**

1. Jenkinson M, Beckmann CF, Behrens TEJ, Woolrich MW, Smith SM (2012): FSL. *NeuroImage*. 62: 782–790.

2. Friedman JH (n.d.): Greedy function approximation: a gradient boosting machine. *The Annals of Statistics*. Vol. 29: 1189–1232.

3. Shen X, Tokoglu F, Papademetris X, Constable RT (2013): Groupwise whole-brain parcellation from resting-state fMRI data for network node identification. *NeuroImage*. 82: 403–415.

4. Finn ES, Shen X, Scheinost D, Rosenberg MD, Huang J, Chun MM, *et al.* (2015): Functional connectome fingerprinting: identifying individuals using patterns of brain connectivity. *Nature Publishing Group*. 18: 1664–1671.

5. Barron DS, Fox PT (2015): *BrainMap Database as a Resource for Computational Modeling*. *Brain Mapping: An Encyclopedic Reference*. Elsevier Inc., pp 675–683.

6. Lancaster JL, Laird AR, Eickhoff SB, Martinez MJ, Fox PM, Fox PT (2012): Automated regional behavioral analysis for human brain images. *Frontiers in Neuroinformatics*. 6: 23.

7. Laird AR, Fox PM, Eickhoff SB, Turner JA, Ray KL, McKay DR, *et al.* (2011): Behavioral Interpretations of Intrinsic Connectivity Networks. *Journal of Cognitive Neuroscience*. 23: 4022–4037.

8. Barron DS, Eickhoff SB, Clos M, Fox PT (2015): Human pulvinar functional organization and connectivity. *Hum Brain Mapp*. 36: 2417–2431.

Supplementary Table & Figure Captions:

**Supplementary Figure 1: Outline of Classifier Type and Cross-Validation Strategy**. FvH=fear versus happy COPE contrast; HvF=happy versus fear COPE contrast (a mathematical inverse of FvH); FvI=fear versus inter-task (fixation) COPE contrast; HvI=happy versus inter-task (fixaton) COPE contrast; FvH_drug_=FvH for subjects administered drug; FvH_placebo_=FvH for subjects administered placebo.

**Supplementary Figure 2a. Accuracies for the two emotional valence discriminations**. The fear versus fixation and happy versus fixation contrasts (left) were unable to be reliably discriminated. The fear versus happy and happy versus fixation contrasts (right) were able to be, as described in the results. Studies are organized on a clinical spectrum, from healthy (H), to low neurotic (LN), to high neurotic (HN), to dysphoric (DYS), to major depressive disorder (MDD). The individual studies are presented in the same order as in Figure 2, with the addition of Healthy (representing only healthy subjects) and ALL (representing all subjects) train-test pairings. Numbers are accuracy score and p-value separated by a comma. White lines separate study-level analyses and analyses across healthy-only subjects and all subjects. Green lines indicate significance at respective level: (i) within study classification: no correction for multiple comparisons; (ii) across-study: p<(0.05/10) Bonferroni correction for multiple comparisons; (iii) across all-subjects: no correction for multiple comparisons; (iv) across-all-studies p<(0.05/10) Bonferroni correction for multiple comparisons. Accuracies based on a bimodal distribution test. Shown below are results for the within-study classification (diagonal), across-study classification (off-diagonal), across-all-subjects classification (bottom-right corner, outlined with white), and across-all-studies classification (bottom row, outlined in white).

**Supplementary Figure 2b. Accuracies for the pharmacologic effect discriminations**. Drug and placebo were unable to be reliably discriminated, as described in the results. Studies are organized on a clinical spectrum, from healthy (H), to low neurotic (LN), to high neurotic (HN), to dysphoric (DYS), to major depressive disorder (MDD). ). The individual studies are presented in the same order as in Figure 2, with the addition of Healthy (representing only healthy subjects) and ALL (representing all subjects) train-test pairings. Numbers are accuracy score and p-value separated by a comma. White lines separate study-level analyses and analyses across healthy-only subjects and all subjects. Green lines indicate significance at respective level: (i) within study classification: no correction for multiple comparisons; (ii) across-study: p<(0.05/10) Bonferroni correction for multiple comparisons; (iii) across all-subjects: no correction for multiple comparisons; (iv) across-all-studies p<(0.05/10) Bonferroni correction for multiple comparisons. Accuracies based on a bimodal distribution test. Shown below are results for the within-study classification (diagonal), across-study classification (off-diagonal), across-all-subjects classification (bottom-right corner, outlined with white), and across-all-studies classification (bottom row, outlined in white).

**Supplementary Figure 3A.** Coronal view of 10% highest weighted parcels from the emotional valence classification. Weightings from classifier trained and tested on healthy subjects are shown in red; weightings trained and tested on dysphoric and MDD subjects are shown in green. Yellow represents overlap of the two, centered in the fusiform gyrus.

**Supplementary Figure 3B.** Coronal view of 10% highest weighted parcels from the emotional valence classification. Weightings from classifier trained and tested on healthy subjects are shown in red; weightings trained and tested on dysphoric and MDD subjects are shown in green. Yellow represents overlap of the two, centered in the fusiform gyrus.

**Supplementary Figure 4.** Laterality of strongest 10% of parcel weightings. For the healthy subjects (left figure), weightings were lateralized to the left hemisphere. For the dysphoric and MDD subjects, the highest weighting were lateralized to the right hemisphere.

Supplementary Figure 1.


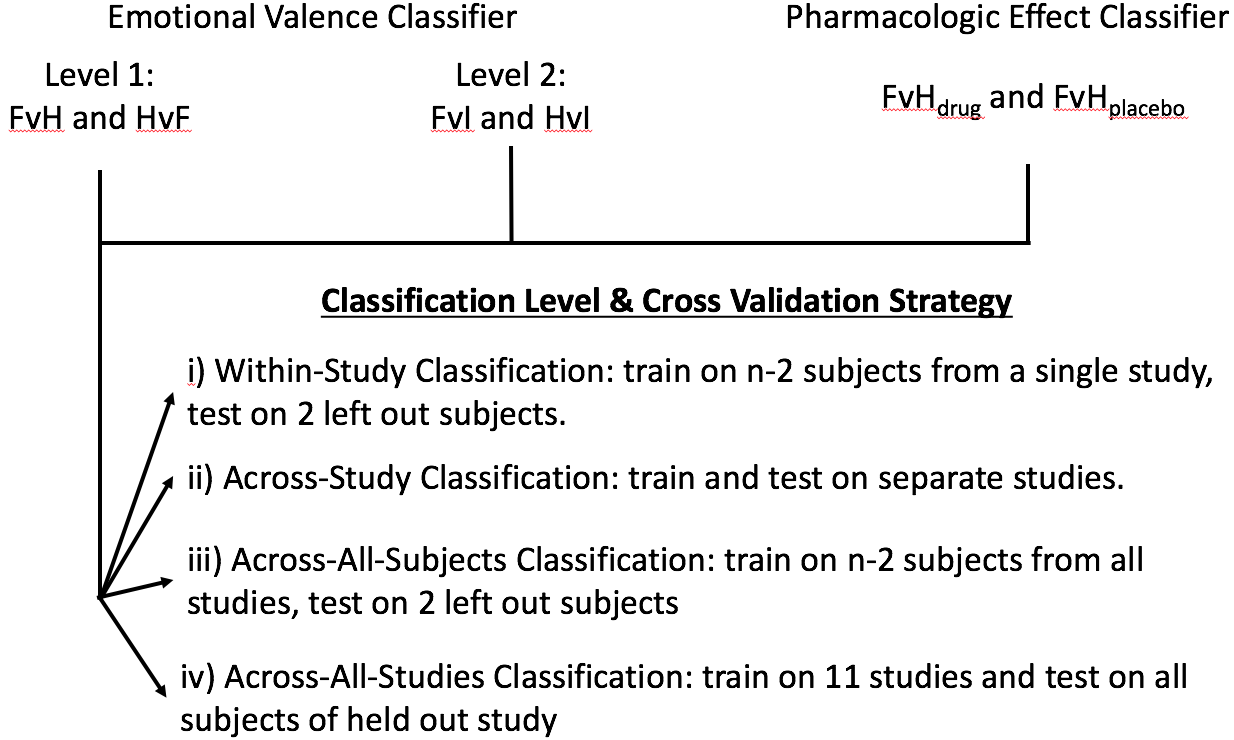


Supplementary Figure 2a.


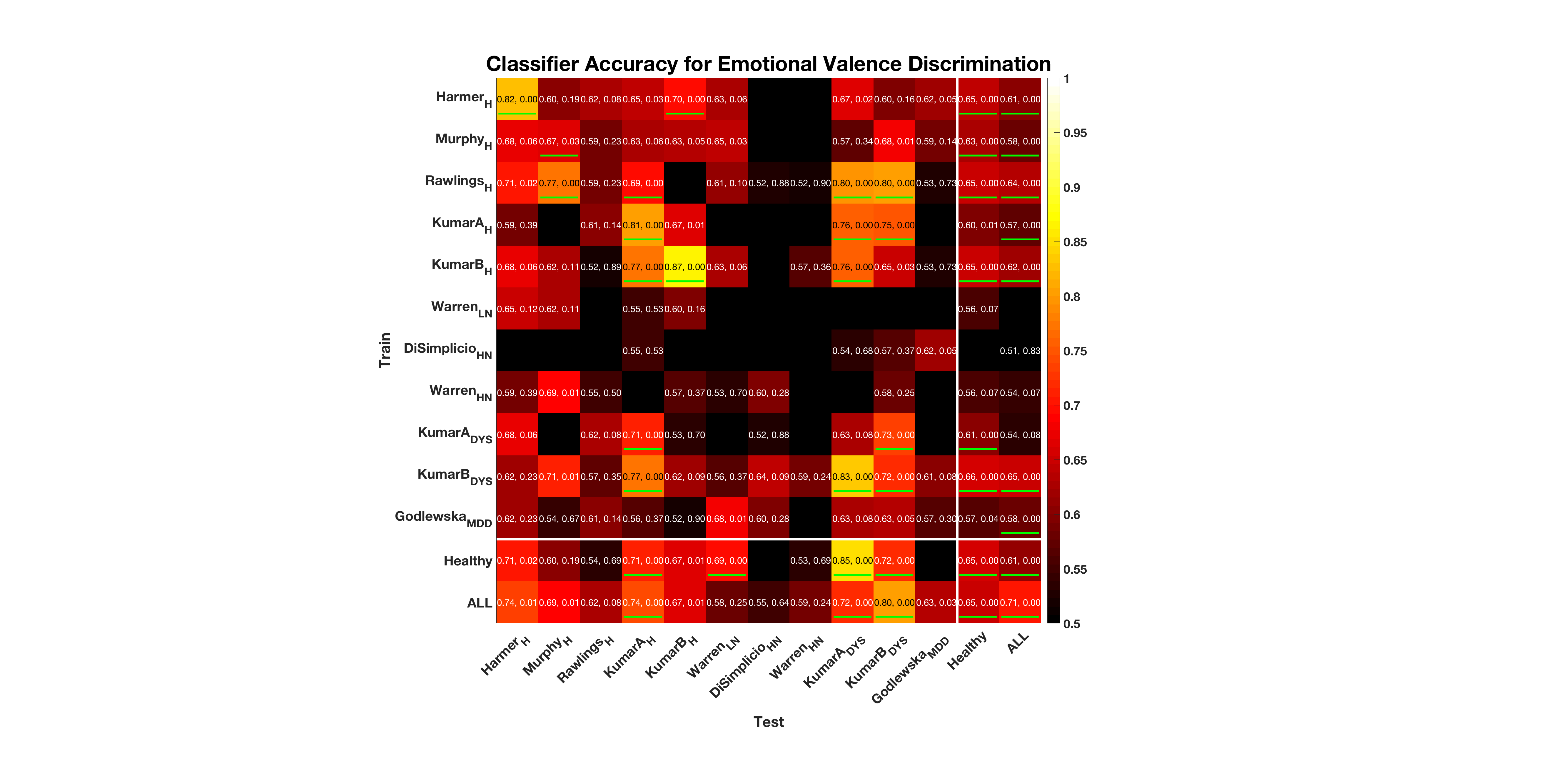

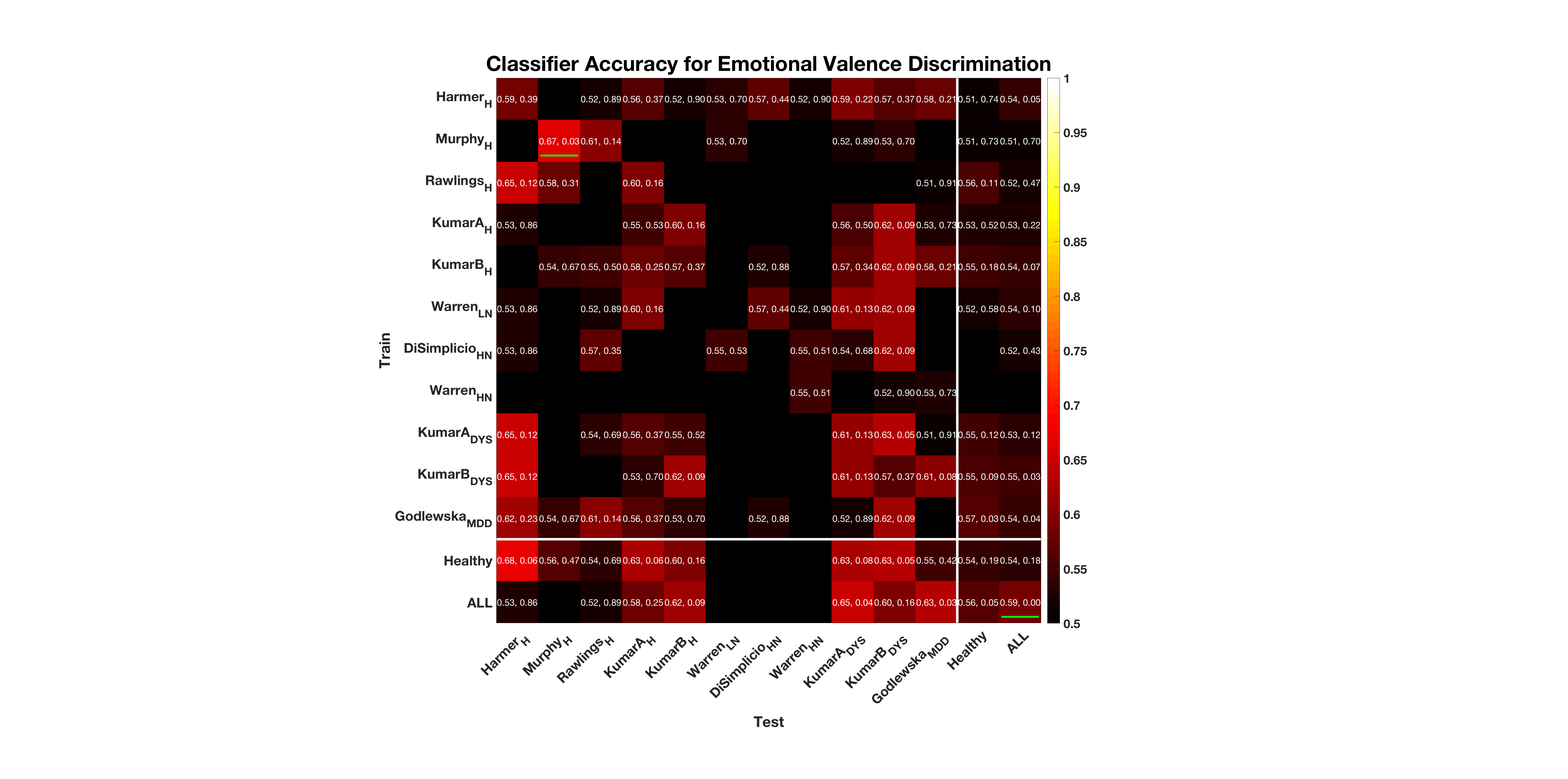


Supplementary Figure 2b.


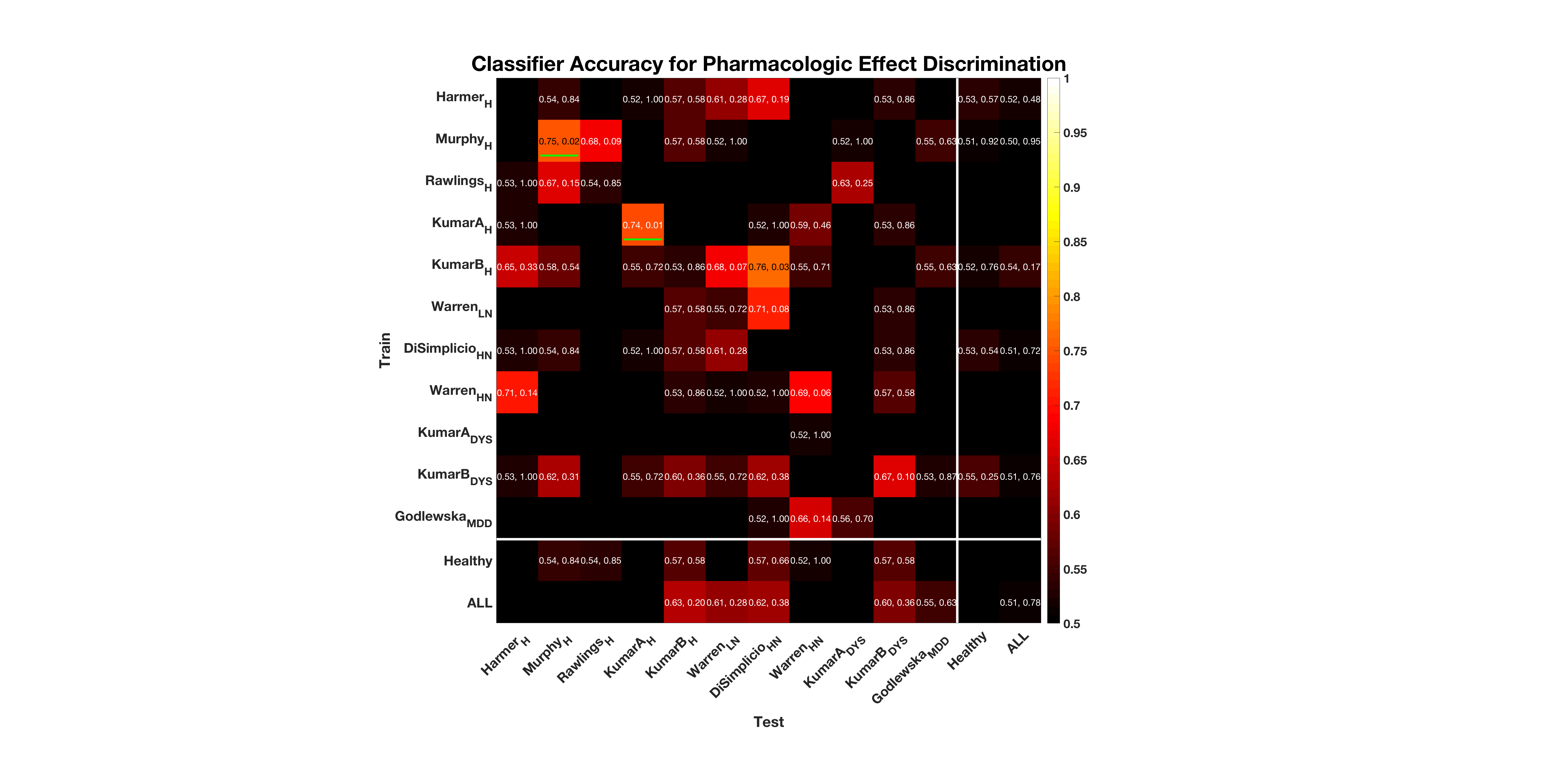


Supplementary Figure 3a.


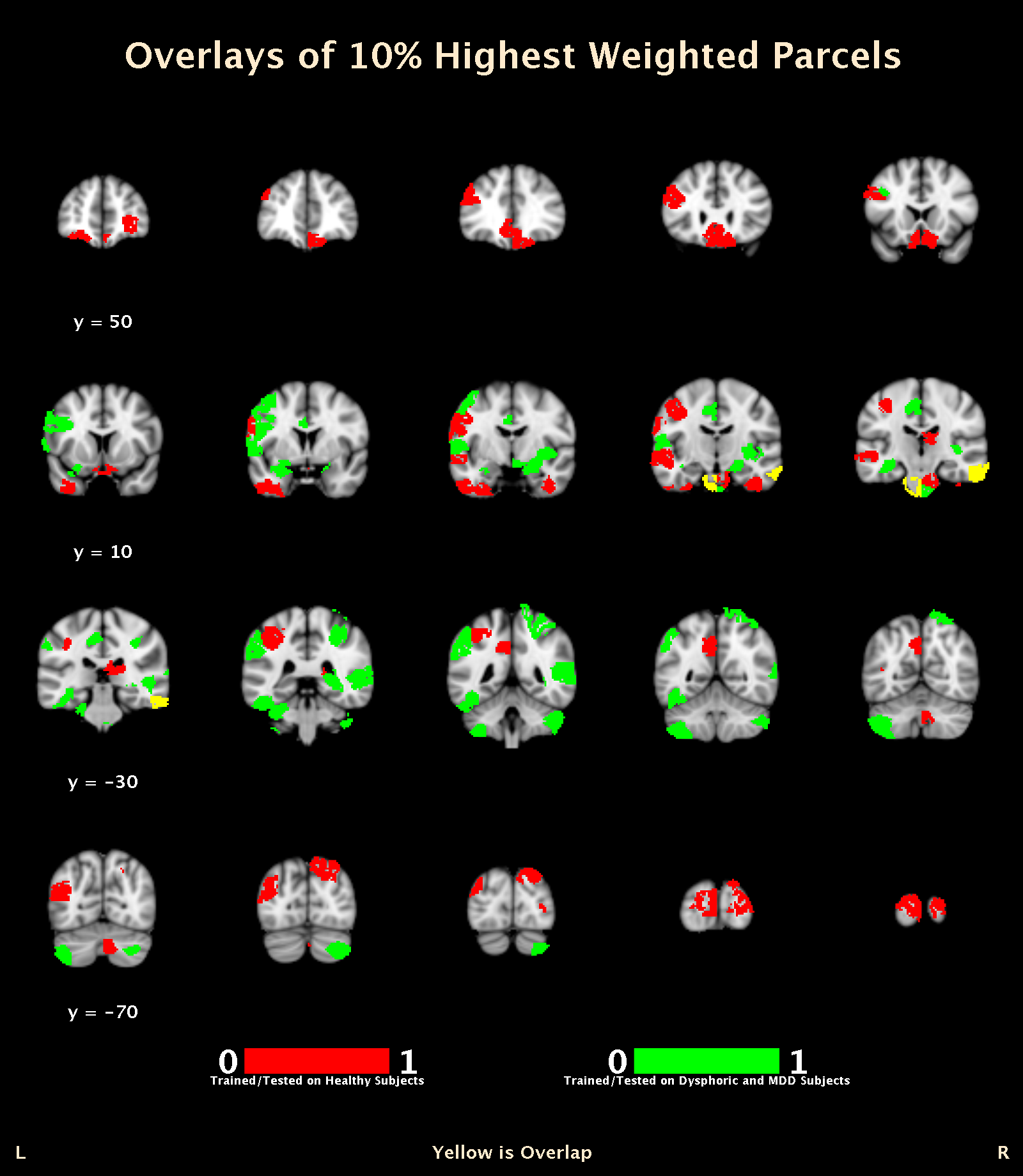


Supplementary Figure 3b.


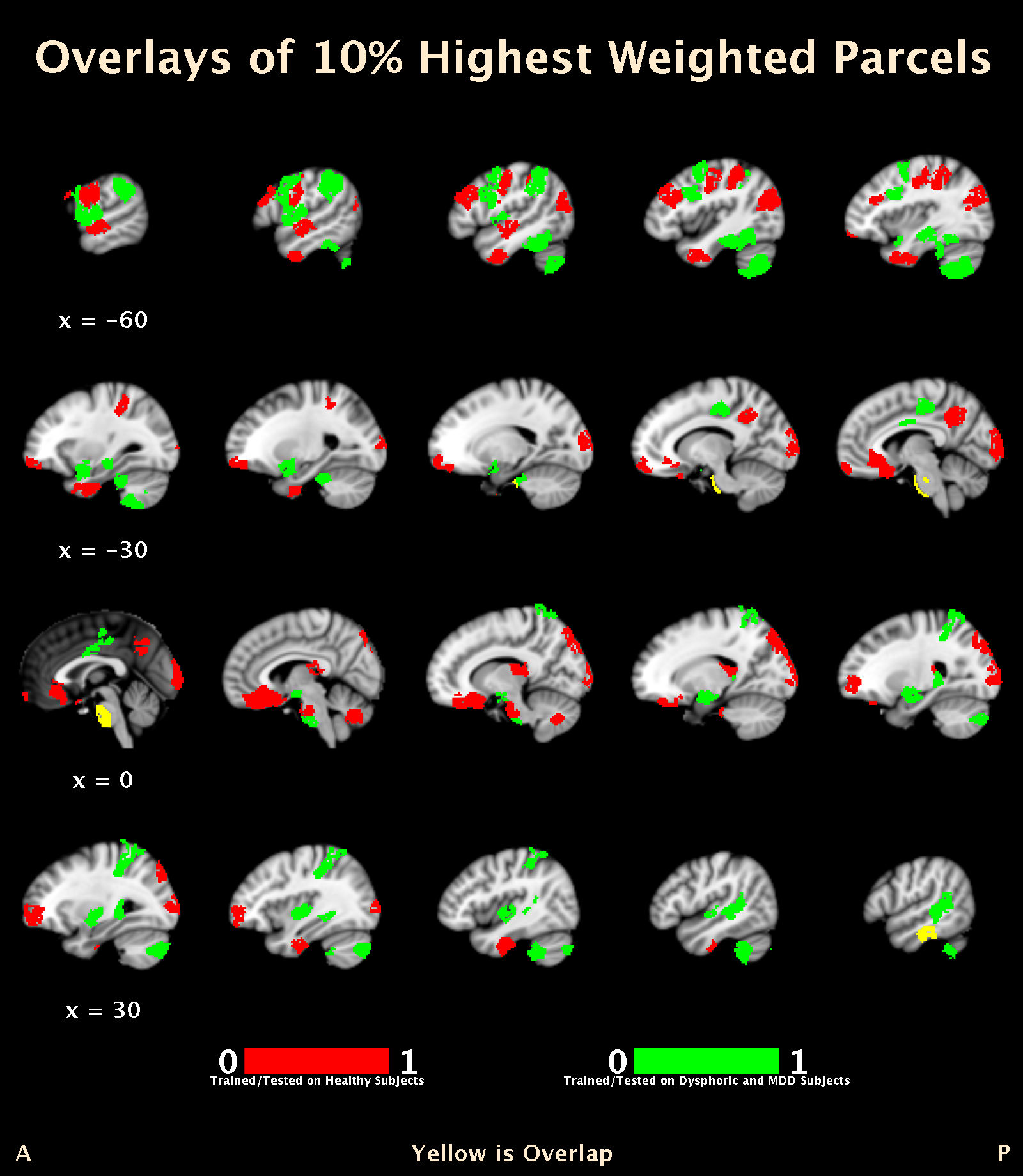


Supplementary Figure 4.


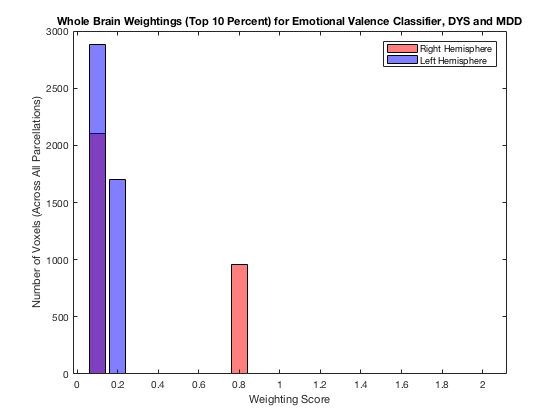

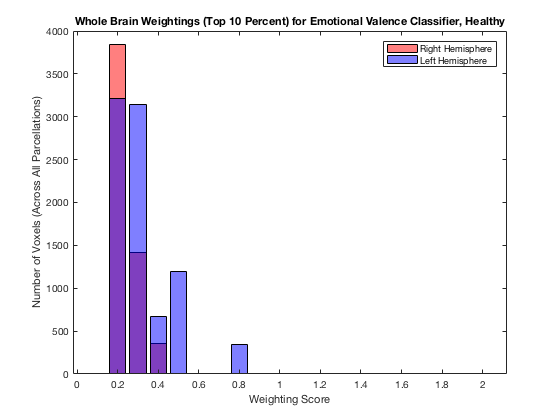

Supplement: Supplementary file 1 — Supplementary material [file mmc1.docx]
